# Circulating B-Vitamins and Smoking Habits Are Associated with Serum Polyunsaturated Fatty Acids in Patients with Suspected Coronary Heart Disease: A Cross-Sectional Study

**DOI:** 10.1371/journal.pone.0129049

**Published:** 2015-06-03

**Authors:** Eli Skeie, Elin Strand, Eva R. Pedersen, Bodil Bjørndal, Pavol Bohov, Rolf K. Berge, Gard F. T. Svingen, Reinhard Seifert, Per M. Ueland, Øivind Midttun, Arve Ulvik, Steinar Hustad, Christian A. Drevon, Jesse F. Gregory, Ottar Nygård

**Affiliations:** 1 Department of Heart Disease, Haukeland University Hospital, Bergen, Norway; 2 Department of Clinical Science, University of Bergen, Bergen, Norway; 3 Bevital AS, Laboratory building, Bergen, Norway; 4 Department of Nutrition, Institute of Basic Medical Sciences, University of Oslo, Oslo, Norway; 5 Food Science and Human Nutrition Department, University of Florida, Gainesville, Florida, United States of America; CSIR-INSTITUTE OF GENOMICS AND INTEGRATIVE BIOLOGY, INDIA

## Abstract

The long-chain polyunsaturated fatty acids are considered to be of major health importance, and recent studies indicate that their endogenous metabolism is influenced by B-vitamin status and smoking habits. We investigated the associations of circulating B-vitamins and smoking habits with serum polyunsaturated fatty acids among 1,366 patients who underwent coronary angiography due to suspected coronary heart disease at Haukeland University Hospital, Norway. Of these, 52% provided information on dietary habits by a food frequency questionnaire. Associations were assessed using partial correlation (Spearman’s rho). In the total population, the concentrations of most circulating B-vitamins were positively associated with serum n-3 polyunsaturated fatty acids, but negatively with serum n-6 polyunsaturated fatty acids. However, the associations between B-vitamins and polyunsaturated fatty acids tended to be weaker in smokers. This could not be solely explained by differences in dietary intake. Furthermore, plasma cotinine, a marker of recent nicotine exposure, showed a negative relationship with serum n-3 polyunsaturated fatty acids, but a positive relationship with serum n-6 polyunsaturated fatty acids. In conclusion, circulating B-vitamins are, in contrast to plasma cotinine, generally positively associated with serum n-3 polyunsaturated fatty acids and negatively with serum n-6 polyunsaturated fatty acids in patients with suspected coronary heart disease. Further studies should investigate whether B-vitamin status and smoking habits may modify the clinical effects of polyunsaturated fatty acid intake.

## Introduction

The n-3 long-chain polyunsaturated fatty acids [LCPUFAs^5^: EPA, docosapentaenoic acid (DPA) and DHA] are proposed to have several beneficial health effects [1]. The human body is able to synthesize most fatty acids, except for the n-3 alpha-linolenic acid (ALA) and the n-6 linoleic acid (LA), which must be obtained from foods or supplements [1]. These essential polyunsaturated fatty acids (PUFAs) can be further metabolized into n-3 and n-6 LCPUFAs, but in limited amounts, partly due to the competition for the same enzymes (elongases and desaturases) in their conversion [1]. Because of this internal competition and the importance of generating sufficient amounts of n-3 LCPUFAs, the Nordic recommendations specify that at least one percent of the energy intake should be from n-3 PUFAs [2]. Interestingly, recent research indicates that the endogenous metabolism of LCPUFAs might be influenced by other lifestyle and dietary factors in addition to dietary intake of the essential fatty acids. A recent study showed that an induced marginal vitamin B_6_ deficiency in humans leads to a reduction in plasma concentrations of both n-3 and n-6 LCPUFAs [3]. Moreover, studies in animals and cell cultures have shown that certain B-vitamins, including folate, vitamin B_6_ and cobalamin (vitamin B_12_), affect the circulating PUFA profile [4–6].

Interestingly, smoking is associated with lower concentrations of both circulating B-vitamins [7,8] and LCPUFAs [9–11], where dietary inequalities alone do not explain this. N-3 LCPUFAs are considered to be particularly beneficial in relation to cardiovascular disease (CVD) [12], and more knowledge on the determinants of PUFA status should be generated in patients at high risk of CVD. Thus, we investigated the association between circulating concentrations of B-vitamins [folate, riboflavin (vitamin B_2_), pyridoxal 5’-phosphate (vitamin B_6_) and vitamin B_12_], and smoking habits with serum PUFAs in a cross-sectional study among 1,366 patients with suspected coronary heart disease (CHD).

## Materials and Methods

### Study population

The Bergen Coronary Angiography Cohort (BECAC) includes an unselected cohort of 4,241 adult patients (> 98% white) who underwent coronary angiography for suspected CHD during the period 2000–2004 at Haukeland University Hospital (Bergen, Norway). The present substudy includes the initial 1,366 BECAC patients recruited during 2000–2001, of whom 709 (52%) also participated in the Western Norway B Vitamin Intervention Trial (WENBIT) [13].

A signed consent form was obtained from all participants. The study was approved by the Regional Committee for Medical and Health Research Ethics and the Norwegian Data Inspectorate.

### Assessment of clinical and dietary data

A self-administered questionnaire completed by each patient was used to collect information about medical history, risk factors and medications, and was checked against medical records as previously reported [13]. Fasting was defined as not having ingested any food or beverages at least 6 hours prior to blood sampling, and hypercholesterolemia was defined as serum total cholesterol ≥ 6.5 mmol/L. Smokers included self-reported current smokers, those who had quit smoking within less than one month prior to baseline, and patients with plasma cotinine ≥ 85 nmol/L [14]. The majority of participants recruited in WENBIT were asked to complete a semiquantitative food-frequency questionnaire (FFQ) with 169 food items at trial enrollment, providing information on dietary habits and use of supplements during the last year [15].

### Analysis of biochemical data

Blood samples were collected prior to coronary angiography, serum/plasma was separated and stored at -80°C until analysis. Serum fatty acid methyl esters were analyzed by gas-liquid chromatography (GC 8000 TOP, Finnigan, USA) on DB1-ms capillary column (j & W Scientific, USA) and quantified as previously described [16]. Fatty acids are given as percentage of total fatty acids in serum. The delta-5-desaturase (D5D) and delta-6-desaturase (D6D) activity indexes were calculated as the concentration of products divided by precursors: n-3 D5D, EPA/eicosatetraeonic acid (ETA); n-3 D6D, stearidonic acid (SDA)/ALA; n-6 D5D, arachidonic acid (AA)/dihomo-γ-linolenic acid (DGLA); n-6 D6D, γ-linolenic acid (GLA)/LA. The omega-3 index [17] was modified to represent the sum of serum EPA and DHA as a fraction of total fatty acids, and we used the EPA/AA-ratio [18] to present a n-3/n-6 PUFA ratio. Plasma concentrations of vitamin B_2_, vitamin B_6_ and cotinine were analyzed by high performance liquid chromatography-tandem mass spectrometry (HPLC-MS/MS) [19] and serum concentrations of folate [20] and vitamin B_12_ [21] were determined by microbiological methods. Plasma concentration of total homocysteine and methylmalonic acid (MMA), a marker of vitamin B_12_ status, were measured using gas chromatography-tandem mass spectrometry (GC-MS/MS) [22]. B-vitamins, homocysteine, MMA and cotinine were analyzed by BEVITAL AS, Bergen, Norway (http://www.bevital.no/).

### Statistical methods

Analyses were conducted in the total population and in non-smokers and smokers separately. Summary measures for continuous variables are reported as medians (25^th^, 75^th^ percentiles) and categorical variables are reported as counts (percentages). The Kolmogorov-Smirnov test was used to assess normality of continuous variables. Mann-Whitney U and Chi-square tests were used to compare characteristics in smokers versus non-smokers as appropriate. Associations between continuous variables were assessed using partial non-parametric correlation (Spearman). Statins are suspected to modify the beneficial CVD effect of n-3 PUFA supplement [23]. Thus ranked values of effective statin dose [expected percent LDL-cholesterol reduction, based on type and dose of specified statin use [24,25] (ordinal)] was included together with age (continuous), gender, dietary intake of n-3 or n-6 PUFA (g/day), plasma vitamin B_2_, vitamin B_6_ and MMA (continuous), and serum folate and vitamin B_12_ (continuous) in the multivariate-adjusted correlation analysis. Further adjustments for BMI and blood glucose did not have any impact on the associations and were thus not included in the final model. Generalized additive models (GAM) were used to describe non-linear associations and were either adjusted for age and gender (simple) or the same covariates as those included in the multivariate correlation analyses. Unadjusted interaction analyses were used to assess differences in associations between non-smokers and smokers.

All probability values were 2-tailed and p-values < 0.05 were considered as statistically significant when comparing nonsmokers and smokers, whereas p-values < 0.01 were considered statistically significant in the multivariate correlations analysis. SPSS software version 21.0 was used for most statistical analysis (SPSS Inc., Chicago, IL, USA). R version 2.15.2 (The R Foundation for Statistical Computing, Vienna, Austria) with package “mgcv” was used to generate GAM curves and to conduct interaction analyses.

## Results

### Characteristics of participants

General characteristics of the total population (n = 1,366) and subgroups of non-smokers (n = 907) and smokers (n = 459) are shown in Table 1. Overall, 74.7% were men and the median (25^th^, 75^th^ percentile) age was 61 (54, 69) years. The majority of the participants had stable angina pectoris (93.3%) and used statins (67.0%). As compared to non-smokers, smokers were younger, had lower BMI, a lower prevalence of diabetes mellitus (both type 1 and 2), lower Troponin T, but more frequently impaired left ventricular ejection fraction.

**Table 1:** Baseline characteristics of participants regarding medical history, risk factors and medications^1^.

	Total	Non-smokers	Smokers	
Characteristics	*(n = 1366)*	*(n = 907)*	*(n = 459)*	*P* ^*2*^
Demographic and clinical				
Men^3^	1021 (74.7)	661 (72.9)	360 (78.4)	0.03
Age (years)^4^	61.0 (54.0, 69.0)	64.0 (56.0, 71.0)	57.0 (50.0, 63.0)	< 0.001
BMI (kg/m2)^4^	26.2 (24.1, 28.7)	26.4 (24.3, 29.0)	26.0 (23.7, 28.4)	0.01
Fasting^3^	187 (13.7)	115 (12.7)	71 (15.7)	0.15
Plasma cotinine (nmol/L)^4^	1.6 (0.0, 510)	0.0 (0.0, 1.6)	1054 (508, 1583)	< 0.001
Cardiovascular risk factors				
Hypercholesterolemia^3^ ^,^ ^5^	755 (55.3)	503 (55.5)	252 (54.9)	0.71
Diabetes^3^ ^,^ ^6^	140 (10.2)	106 (11.7)	34 (7.41)	0.02
Stable angina pectoris^3^	1274 (93.3)	857 (94.5)	417 (90.8)	0.02
Homocysteine (μmol/L)^4^	10.3 (8.6, 12.3)	10.2 (8.5, 12.3)	10.6 (8.8, 12.4)	0.12
Functional markers for cardiac function				
Left ventricular ejection fraction < 50%^3^	114 (8.3)	65 (7.2)	49 (10.7)	0.027
Troponin T ≥14 ng/L^3^	201 (14.7)	151 (16.6)	50 (10.9)	0.021
Medication^3^ ^,^ ^7^				
Statin	915 (67.0)	610 (67.3)	305 (66.4)	0.68
Acetylsalicylic acid	1075 (78.7)	719 (79.3)	356 (77.6)	0.47
β-blocker	987 (72.3)	679 (74.9)	308 (67.1)	0.002
Calcium channel blocker	305 (22.3)	206 (22.7)	99 (21.6)	0.63
ACE inhibitors	255 (18.7)	190 (20.9)	65 (14.2)	0.002
Angiotensin II receptor antagonist	129 (9.4)	81 (8.9)	48 (10.5)	0.36
Loop diuretics	135 (9.9)	100 (11.0)	35 (7.6)	0.047

^1^ Missing values: Cotinine: n = 3 (0.2%), β-blocker: n = 1 (0.1%); Fasting: n = 74 (5.4%); Hypercholesterolemia: n = 81 (5.9%); Troponin T: n = 170 (12.4%). ACE, angiotensin converting enzyme.

^2^ *P* values for differences between smokers and non-smokers were calculated by using Mann-Whitney U test for continuous variables and chi-square tests for categorical variables.

^3^ n (%).

^4^ Median (25^th^, 75^th^ percentile).

^5^ Serum total cholesterol ≥ 6.5mmol/L.

^6^ Includes diabetes types 1 and 2.

^7^ Medication prior to coronary angiography.

### Dietary intake and circulating concentrations of B-vitamins and PUFAs

Fifty-two percent of the participants completed a FFQ at trial enrollment. A short summary of dietary intake and circulating concentrations of B-vitamins and PUFAs are shown in Table 2 and Table 3, respectively. Even though the dietary intake of B-vitamins did not significantly differ between non-smokers and smokers, there were lower circulating concentrations of folate, vitamin B_2_ and vitamin B_6_ among smokers. Further, smokers had a higher dietary intake of ALA, but lower dietary intake of n-3 LCPUFAs and total serum n-3 PUFAs than non-smokers. Dietary intake of AA did not significantly differ between non-smokers and smokers, but participants who smoked had higher dietary intake of LA and total serum n-6 PUFAs.

**Table 2:** Dietary intake of B-vitamins and fatty acids according to smoking status^1^.

	Total	Non-smokers	Smokers	
Nutrient^2^	*(n = 709)*	*(n = 486)*	*(n = 223)*	*P* ^*3*^
B-vitamins				
Folate (μg/d)	228 (181, 281)	223 (179, 279)	236 (183, 289)	0.21
B2 (mg/d)	1.51 (1.15, 1.93)	1.52 (1.16, 1.90)	1.50 (1.10, 2.01)	0.68
B6 (mg/d)	1.55 (1.19, 1.93)	1.52 (1.18, 1.89)	1.58 (1.98, 1.20)	0.39
B12 (μg/d)	7.50 (5.40, 10.3)	7.50 (5.35, 10.1)	7.90 (5.50, 10.8)	0.21
Saturated fatty acids (g/d)	26.5 (19.4, 34.2)	25.4 (18.7, 32.8)	29.4 (21.1, 37.6)	< 0.001
MUFAs (g/d)	23.1 (17.5, 29.8)	22.6 (16.8, 29.1)	24.8 (18.8, 34.2)	0.001
PUFAs (g/d)				
Total n-3^4^	3.00 (2.21, 4.17)	2.96 (2.20, 4.2)	3.09 (2.30, 4.29)	0.28
ALA	1.85 (1.36, 2.43)	1.76 (1.30, 2.26)	2.05 (1.49, 2.69)	< 0.001
EPA, DPA and DHA	1.05 (0.58, 1.76)	1.13 (0.59, 1.91)	0.92 (0.52, 1.59)	0.03
Total n-6^5^	12.6 (8.91, 16.9)	12.0 (8.71, 15.7)	14.0 (10.3, 19.3)	0.001
LA	12.5 (8.80, 16.7)	11.93 (8.61, 15.6)	13.8 (10.1, 19.2)	< 0.001
AA	0.11 (0.08, 0.14)	0.11 (0.07, 0.14)	0.11 (0.08, 0.15)	0.15

^1^ Of participants who completed the food frequency questionnaire, missing values are: B_6_: n = 3 (0.4%), B_12_: n = 3 (0.4%). AA, arachidonic acid; ALA, alpha-linolenic acid; B_2_, riboflavin; B_6_, pyridoxal 5’-phosphate; B_12_, cobalamin; DPA, docosapentaenoic acid; LA, linoleic acid.

^2^ Median (25^th^, 75^th^ percentile).

^3^ Difference between smokers and non-smokers were calculated by Mann-Whitney U test.

^4^ Sum of ALA, EPA, DPA and DHA.

^5^ Sum of LA and AA.

**Table 3:** Serum/plasma concentrations of vitamins, subgroups of fatty acids, fatty acid indexes and activity index of desaturases according to smoking status^1^.

	Total	Non-smokers	Smokers	
Characteristics	*(n = 1366)*	*(n = 907)*	*(n = 459)*	*P* ^*2*^
B-vitamins and vitamin-marker				
Folate (nmol/L)	10.0 (7.3, 14.6)	10.7 (7.9, 15.2)	8.90 (6.5, 12.5)	< 0.001
B2 (nmol/L)	11.0 (7.4, 17.5)	11.8 (7.8, 18.3)	9.91 (6.8, 15.9)	< 0.001
B6 (nmol/L)	42.1 (29.9, 59.9)	44.1 (32.0, 61.6)	37.5 (25.9, 54.8)	< 0.001
B12 (pmol/L)	351 (269, 443)	353 (270, 444)	346 (266, 436)	0.80
MMA (μmol/L)	0.16 (0.13, 0.20)	0.16 (0.14, 0.21)	0.16 (0.13, 0.20)	0.11
Fatty acids (% of total fatty acids)				
SFA	33.0 (31.9, 34.8)	33.0 (31.9, 34.7)	33.0 (31.6, 34.9)	0.99
MUFA	23.1 (20.7, 25.5)	22.9 (20.8, 25.1)	23.5 (20.5, 26.3)	0.01
n-3 PUFA	7.27 (5.61, 9.35)	7.83 (6.12, 10.0)	6.18 (4.8, 8.09)	< 0.001
n-6 PUFA	35.0 (31.7, 38.7)	34.6 (31.6, 37.9)	35.6 (32.1, 39.6)	0.001
Fatty acid indexes				
Omega-3 index	5.68 (4.14, 7.60)	6.19 (4.59, 8.28)	4.67 (3.40, 6.44)	< 0.001
EPA/AA-ratio^3^	34.5 (20.3, 58.8)	40.2 (23.1, 65.6)	26.1 (15.9, 47.9)	< 0.001
Activity index of desaturases				
n-3 D5D	13.2 (8.97, 19.4)	14.4 (9.88, 20.7)	10.8 (7.29, 16.3)	< 0.001
n-6 D5D	4.33 (3.60, 5.40)	4.39 (3.61, 5.54)	4.30 (3.55, 5.20)	0.10
n-3 D6D	0.05 (0.03, 0.08)	0.05 (0.05, 0.08)	0.04 (0.03, 0.07)	0.001
n-6 D6D	0.01 (0.01, 0.02)	0.01 (0.01, 0.02)	0.01 (0.01, 0.02)	0.10

^1^ Given as median (25^th^, 75^th^ percentile). Missing values: Folate: n = 2 (0.1%); B_2_: n = 2 (0.1%); B_6_: n = 2 (0.1%); B_12_: n = 494 (36.2%); MMA: n = 2 (0.1%). AA, arachidonic acid; B_2_, riboflavin; B_6_, pyridoxal 5’-phosphatase; B_12_, cobalamin; D5D, delta 5 desaturase; D6D, delta 6 desaturase; MMA, methylmalonic acid; omega-3 index, (EPA + DHA) of total fatty acids; n-3 D5D, EPA/eicosatetraeonic acid; n-3 D6D, stearidonic acid/alpha linolenic acid; n-6 D5D, arachidonic acid/dihomo-γ-linolenic acid; n-6 D6D, γ-linolenic acid/ linoleic acid; SFA, saturated fatty acids.

^2^ Difference between smokers and non-smokers were calculated by Mann-Whitney U test.

^3^ EPA/AA-ratio * 100

### Associations between circulating B-vitamins and PUFAs

**Fig 1:**
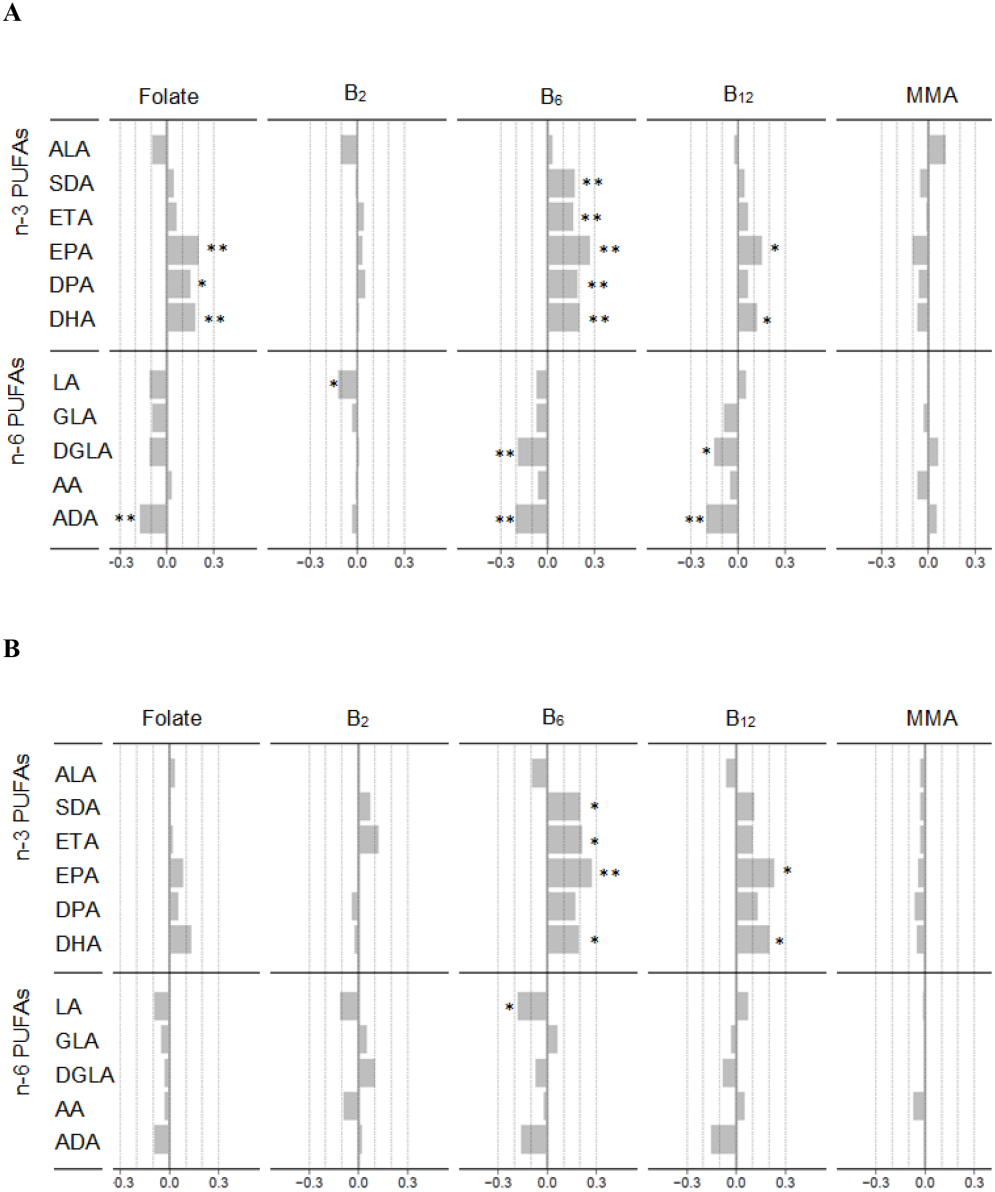
The relationship between circulating B-vitamins and serum n-3 and n-6 PUFAs in non-smokers and smokers. Spearman’s rho (r) of ranked values of circulating B-vitamins folate, B_2_, B_6_, B_12_ and MMA with serum n-3 and n-6 PUFAs in non-smokers (n = 480) **(A)** and smokers (n = 215) **(B)** who completed the food frequency questionnaire. The models for n-3 PUFAs were adjusted for gender, age, effective statin dose and dietary intake of n-3 PUFAs (ALA, EPA, DPA and DHA) (g/d); The models for n-6 PUFAs were adjusted for gender, age, effective statin dose and dietary intake of n-6 PUFAs (LA and AA) (g/d). AA, arachidonic acid; ADA, Adrenic acid; ALA, alpha linolenic acid; B_2_, riboflavin; B_6_, pyridoxal 5’-phosphate; B_12_, cobalamin; DGLA, dihomo-γ-linolenic acid; DPA, docosapentaenoic acid; ETA, eicosatetraeonic acid; GLA, γ-linolenic acid; LA, linoleic acid; MMA, methylmalonic acid; SDA, Stearidonic acid. * p <0.01, ** p < 0.001.

Among non-smokers, simple correlation analysis demonstrated positive associations of circulating folate, vitamins B_6_ and B_12_ with n-3 PUFAs, the omega-3 index, EPA/AA-ratio and n-3 D5D (S1 Table). Furthermore, circulating folate and vitamin B_6_ were negatively related with serum n-6 PUFAs and positively with n-3 D6D and n-6 D5D. None of the associations of serum vitamin B_12_ with fatty acids, fatty acid indexes or activity indexes of desaturases were reflected by a similar inverse relationship of the metabolic vitamin B_12_ marker, MMA. The strongest observed relation was between plasma B_6_ and the EPA/AA-ratio (S1 Table). These associations were essentially confirmed by multivariate analyses, demonstrating circulating B-vitamins to be positively related to serum n-3 PUFAs, and negatively related to serum n-6 PUFAs. The strongest associations were seen for plasma vitamin B_6_, which was directly associated with serum n-3 LCPUFAs and inversely with serum n-6 LCPUFAs, and declined in the order EPA, DHA/ADA and DGLA (Fig 1A).

Further, GAM was used to investigate the dose-response relationship for the strongest associations found by the multivariate correlation analyses among non-smokers for serum n-3 PUFAs (S1 Fig) and n-6 PUFAs (S2 Fig). The positive associations with most serum n-3 PUFAs leveled off at higher vitamin concentrations, whereas the inverse associations with the serum n-6 PUFAs were essentially linear across the whole distribution of circulating folate, vitamin B_6_ and vitamin B_12_ in non-smokers.

In smokers, crude associations were similar to those observed among non-smokers, except for serum folate that did not show any significant association with serum fatty acids, fatty acid indexes or activity indexes of desaturases in this group (S1 Table). Serum folate showed no significant correlation with serum n-3 or n-6 PUFAs in the multivariate analysis (Fig 1B). Most associations of plasma vitamin B_6_ and serum vitamin B_12_ with serum n-3 PUFAs were similar in smokers as compared to non-smokers in the multivariate analysis, whereas the associations with serum n-6 PUFAs tended to be weaker in smokers.

Even though the correlations between circulating B-vitamins and PUFAs tended to be generally weaker in smokers as compared to non-smokers (Fig 1A and 1B), interaction analyses did not detect any significant differences between these subgroups.

### Associations between circulating cotinine and PUFAs

Crude correlation analyses showed strong inverse associations between plasma cotinine and variables containing serum n-3 PUFAs, and a positive, although weaker, association with serum n-6 PUFAs (S1 Table). The multivariate analyses demonstrated plasma cotinine to be inversely associated with all serum n-3 PUFAs [SDA (r = -.11, p = 0.003), ETA (r = -.11, p = 0.004), EPA (r = -.18, p < 0.001), DPA (r = -.25, p < 0.001) and DHA (r = -.24, p < 0.001)], with the exception of ALA. There were no significant associations between plasma cotinine and serum n-6 PUFAs in these analyses.

**Fig 2:**
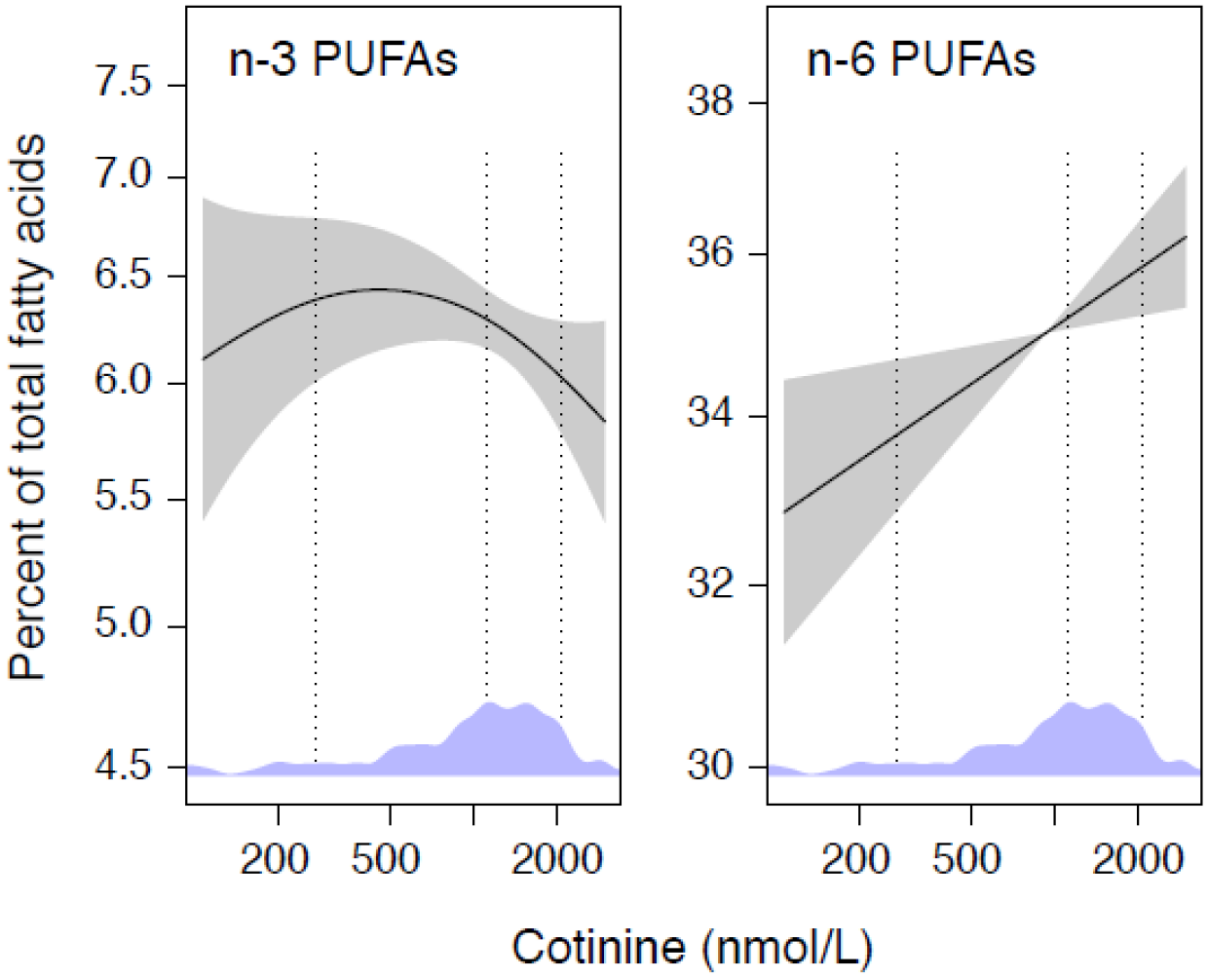
The relationship between cotinine and serum n-3 and n-6 PUFAs. The dose-response relationships between concentrations of plasma cotinine levels (nmol/L) and serum PUFAs (% of total fatty acids) in smokers (n = 459). Associations were modeled by GAM and adjusted for gender and age. Shaded areas indicate 95% confidence intervals. The y-axis spans 2 standard derivations of each outcome. Density plot for the distribution of cotinine are included in diagrams with 10^th^, 50^th^ and 90^th^ percentile marked by dotted, vertical lines.

Among smokers, the multivariate analyses did not show any significant associations between plasma cotinine and serum n-3 PUFAs, but demonstrated a positive association between plasma cotinine and the essential n-6 PUFA, LA in serum (r = .20, p = 0.004). These relationships were further investigated in GAM-curves which demonstrated plasma cotinine to be inversely associated with total serum n-3 PUFAs at cotinine concentrations above ~500 nmol/L, and directly associated with serum n-6 PUFAs throughout the cotinine range (Fig 2).

## Discussion

### Principal findings

In this large cross-sectional study among patients with suspected CHD, we demonstrated that circulating B-vitamins are generally positively related with serum n-3 PUFAs, and negatively related with serum n-6 PUFAs, independent of smoking status. Overall, the strongest association was between plasma vitamin B_6_ and serum EPA. Circulating cotinine, a marker of recent nicotine exposure, tended to be inversely related to serum n-3 PUFAs, and directly related to serum n-6 PUFAs.

### Clinical relevance

Our results indicate that there may be an interaction between B-vitamin status, smoking and circulating PUFA status, which are factors associated with risk of CVD [17,18,26–29]. No beneficial effect of treatment with folic acid/B_12_ or B_6_ was found in WENBIT [13]. Notably, we recently observed that a high dietary intake of n-3 LCPUFAs was associated with an increased risk of fatal myocardial infarction in patients without impairment of glucose metabolism, but with a reduced risk in patients with diabetes [30]. Furthermore, in contrast to results from older studies, recent randomized controlled trials do not find a protective effect of n-3 PUFA supplementation on CVD risk in the general population [31] or in individuals at high risk of cardiovascular events [32]. Moreover, the prescription of statins has increased during the last decade and such treatment may modify the effect of n-3 PUFAs on incident major cardiovascular events [23]. Adjustment for statin treatment did not influence the current results. In addition, a lower prevalence of smokers during the last decades may have contributed to an increase in the circulating concentration of n-3 PUFAs in the general population. This may further limit the possibility to detect beneficial effects of n-3 PUFA supplementation in relation to CVD. Our data should motivate subgroup analysis of B-vitamin status and/or smoking habits when exploring results from studies with PUFA or statin treatment.

### Possible mechanisms

Circulating concentrations of PUFAs are related to the dietary intake of corresponding fatty acids [30]. Moreover, a study in healthy men demonstrated that an increased dietary intake of LA resulted in decreased plasma EPA and increased plasma n-6 eicosadienoic acid (a conversion product of LA), but no changes of plasma AA [33]. The authors suggested that an increased conversion of LA inhibited the ALA conversion to EPA [33]. This may be seen in context with smokers in our study who had a higher dietary intake of LA and a lower circulating EPA/AA-ratio than non-smokers. A low EPA/AA-ratio may also be associated with an increased production of AA generated pro-inflammatory eicosanoids, as opposed to anti-inflammatory eicosanoids which can be derived from EPA [34].

Fatty acids, including PUFAs, are incorporated into cellular phospholipids where important biological functions are exerted. Phosphatidylcholine (PC) is the most abundant group of phospholipids and can be synthesized from two different pathways. The most common pathway is the CDP-choline pathway, while the alternative route goes through the phosphatidylethanolamine (PE) methylation pathway. The latter includes the activity of phosphatidylethanolamine-*N*-methyltransferase (PEMT) to convert PE into PC via sequential methylation reactions [35]. This pathway has been reported to affect the circulating concentration of LCPUFAs in serum, plasma or erythrocytes [4,36,37]. Since PEMT seems to prefer LCPUFA PE as substrate, PC synthesized through this pathway contain more LCPUFA as compared to PC synthesized through the alternative CPD-choline pathway, which is composed of more saturated fatty acids [36]. An increased generation of PC through the PEMT pathway may thus increase the availability of LCPUFA to the peripheral tissue [37]. A study in mice showed that low PEMT activity led to reduced DHA in plasma PC, but an accumulation of DHA in hepatic PE, possibly demonstrating the role of PEMT in mobilization of DHA from liver into plasma [37]. Interestingly, homocysteine, an established risk marker for CVD [38] is formed from S-adenosylhomocysteine which inhibits most methyltransferases, including PEMT. Circulating concentrations of folate, vitamin B_6_ and vitamin B_12_ are inversely associated with that of homocysteine [38]. Thus, these homocysteine-lowering vitamins are suspected to be positively correlated with PEMT activity, and therefore also with the circulating concentrations of LCPUFA [4]. Accordingly, a reduced PEMT activity has been demonstrated in liver microsomes of vitamin B_6_-deficient rats [39]. In further support of this hypothesis, an inverse relationship between circulating homocysteine and DHA is observed in both animal and human studies [4,40,41]. Furthermore, serum AA was positively associated while hepatic AA was negatively associated to vitamin B supplementation in rats on an energy restricted diet [42]. Thus, B-vitamins may indirectly affect the circulating concentration of PUFAs through the hepatic PEMT activity. In the current study we did not measure hepatic PEMT activity, but we observed a positive relationship between the circulating concentrations of folate, vitamin B_6_ and vitamin B_12_ with serum n-3 LCPUFAs.

PUFA status may also be modified through desaturase activity, which introduces new double-bonds to the PUFAs during their conversion into LCPUFAs [1]. Low serum concentrations of vitamin B_6_ are associated with lower estimated D5D and D6D activity indexes in healthy people [43], and vitamin B_6_-deficiency has shown to decrease the activity index of D6D in rat liver microsomes [44]. Moreover, high levels of vitamin B_6_ have been reported to increase both *D5D-* and *D6D* mRNA levels in HepG2 cells [5]. These results may suggest that folate and vitamins B_6_ and B_12_ influence genes and enzymes involved in the metabolism of LCPUFA. In agreement with these experimental data, we demonstrated that the association between circulating B-vitamins and LCPUFAs was stronger than with the essential PUFAs [ALA and LA] in non-smokers. Furthermore, smoking has been reported to decrease the activity indexes of D5D and D6D in previous cell studies [10,11], and further reduce the LA conversion [11]. Our results are in line with these experimental findings in that plasma cotinine is inversely associated with the estimated activity indexes of D5D and D6D, but positively related to serum LA in smokers. Of note, previous studies present the activity indexes of desaturases calculated from n-6 PUFAs [10,11]. In the current study plasma cotinine was only significantly associated with the activity indexes of desaturases when calculated from n-3 PUFAs. However, a decreased activity of D5D and D6D due to smoking may explain the tendency of weaker associations of circulating B vitamins with serum n-3 and n-6 LCPUFA in smokers as compared to non-smokers. Moreover, different single-nucleotide polymorphisms of genes regulating the desaturase enzymes may also affect the PUFA profile [45]. Unfortunately, the present study does not include such information.

Since smoking has both short- and long-term effects on circulating B-vitamins [8], it may also indirectly affect the PUFA profile. Smoking induces systemic oxidative stress in humans [46], whereas folate, vitamin B_2_ and vitamin B_6_ have antioxidant properties [47–49]. Furthermore, smoking is associated with low muscle mass [50]. Muscles are a major depot of vitamin B_6_ [51], and a depletion of these depots may reduce circulating vitamin B_6_. In accordance to a tendency of higher oxidative stress and reduced muscle mass among smokers, we observed them to have lower concentrations of circulating folate, B_2_ and B_6_ as compared to non-smokers.

Taken together, both the current study and results from prior experimental studies suggest a complex metabolic interplay between B-vitamins, PUFAs and smoking status, which may further interfere with the progression of CHD.

### Strengths and limitations

Strengths of the current study include its large, well-characterized population. Data from the FFQ made it possible to adjust for dietary PUFAs when investigating the associations of B-vitamins and cotinine with circulating PUFAs. Further, plasma cotinine was used to investigate the dose-response relationship between nicotine exposure and PUFAs.

There were some limitations of the data collection. Most patients were not fasting, which may influence some of the measurements, including the fatty acid composition. Of note, the circulating concentrations of n-3 PUFAs, n-6 PUFAs, folate, B_2_, B_12_ and cotinine did not differ between fasted and non-fasted participants, whereas plasma B_6_ was lower in those who fasted. However, no effect of prandial status on plasma vitamin B_6_ is reported (www.bevital.no). Furthermore, there were no differences in the prevalence of fasting between non-smokers and smokers. Since both cigarette smoke [14] and smokeless tobacco [52] affects the circulating cotinine concentration, subjects using smokeless tobacco may have been misclassified as smokers. However, most of the subjects (approximately 85%) confirmed through self-report that they were current smokers or had been smokers within the past month before included in the study. Moreover, only subjects enrolled in WENBIT were asked to fill in the FFQ, introducing a potential for selection bias. Respondents of the FFQ were more often non-smokers and had a higher frequency of stable angina pectoris. Since patients with known CHD may be more aware of their dietary intake, this may influence the results. However, the median dietary intake of n-3 PUFAs and B-vitamins in our study population was comparable with intakes observed in the general population from the same region using the same questionnaire [53]. Furthermore, our data are based on single measurements at baseline, which may lead to underestimation of the true strength of the associations, due to regression dilution bias [54].

## Conclusions

In conclusion, we demonstrate that circulating B-vitamins and smoking habits are associated with serum PUFAs in patients with suspected CHD. These associations should be evaluated in general populations, and motivate further studies on interactions between B-vitamins, smoking status and lipid metabolism in relation to lifestyle diseases.

## Supporting Information

S1 Fig The relationship between selected circulating B-vitamins and serum n-3 PUFAs. The dose-response relationships between concentrations of serum folate, plasma vitamin B_6_ and serum vitamin B_12_ with concentrations of serum EPA and DHA in non-smokers (n = 480). Associations were modeled by GAM adjusted for age, gender, effective statin dose and dietary intake of n-3 PUFAs. Shaded areas indicate 95% confidence intervals. The y-axis spans 2 standard derivations of each outcome. Density plots for the distribution of B-vitamins are included in diagrams with 10^th^, 50^th^ and 90^th^ percentiles marked by dotted, vertical lines. B_6_, pyridoxal 5’-phosphate; B_12_, cobalamin.
(TIF)

S2 Fig The relationship between selected circulating B-vitamins and serum n-6 PUFAs. The dose-response relationships between concentrations of serum folate, plasma vitamin B_6_ and serum vitamin B_12_ with concentrations of serum DGLA and ADA in non-smokers (n = 480). Associations were modeled by GAM adjusted for age, gender, effective statin dose and dietary intake of n-6 PUFAs. Shaded areas indicate 95% confidence intervals. The y-axis spans 2 standard derivations of each outcome. Density plot for the distribution of B-vitamins are included in diagrams with 10^th^, 50^th^ and 90^th^ percentiles marked by dotted, vertical lines. DGLA, dihomo-γ-linolenic acid; ADA, Adrenic acid; B_6_, pyridoxal 5’-phosphate; B_12_, cobalamin.
(TIF)

S1 Table Bivariate correlations of plasma cotinine and circulating B-vitamins with serum concentrations of fatty acids, fatty acid indexes and activity indexes of desaturases^1^. ^1^ Values are given as Spearman’s rho. B_2_, riboflavin; B_6_, pyridoxal 5’- phosphate; B_12_, cobalamin; MMA, methylmalonic acid; omega-3 Index, (EPA + DHA) of total fatty acids; AA, arachidonic acid; D5D, delta 5 desaturase; n-3 D5D, EPA/eicosatetraenoic acid; n-6 D5D, arachidonic acid/dihomo-γ-linolenic acid; D6D, delta 6 desaturase; n-3 D6D, stearidonic acid/alpha linolenic acid; n-6 D6D, γ-linolenic acid/ linoleic acid. * p < 0.01, ** p < 0.001.
(TIF)
