# Supplementary figures and images for: Circulating B-Vitamins and Smoking Habits Are Associated with Serum Polyunsaturated Fatty Acids in Patients with Suspected Coronary Heart Disease: A Cross-Sectional Study
Source: PLoS One. 2015 Jun 3;10(6):e0129049. doi: 10.1371/journal.pone.0129049 (PMC4454679; doi:10.1371/journal.pone.0129049)

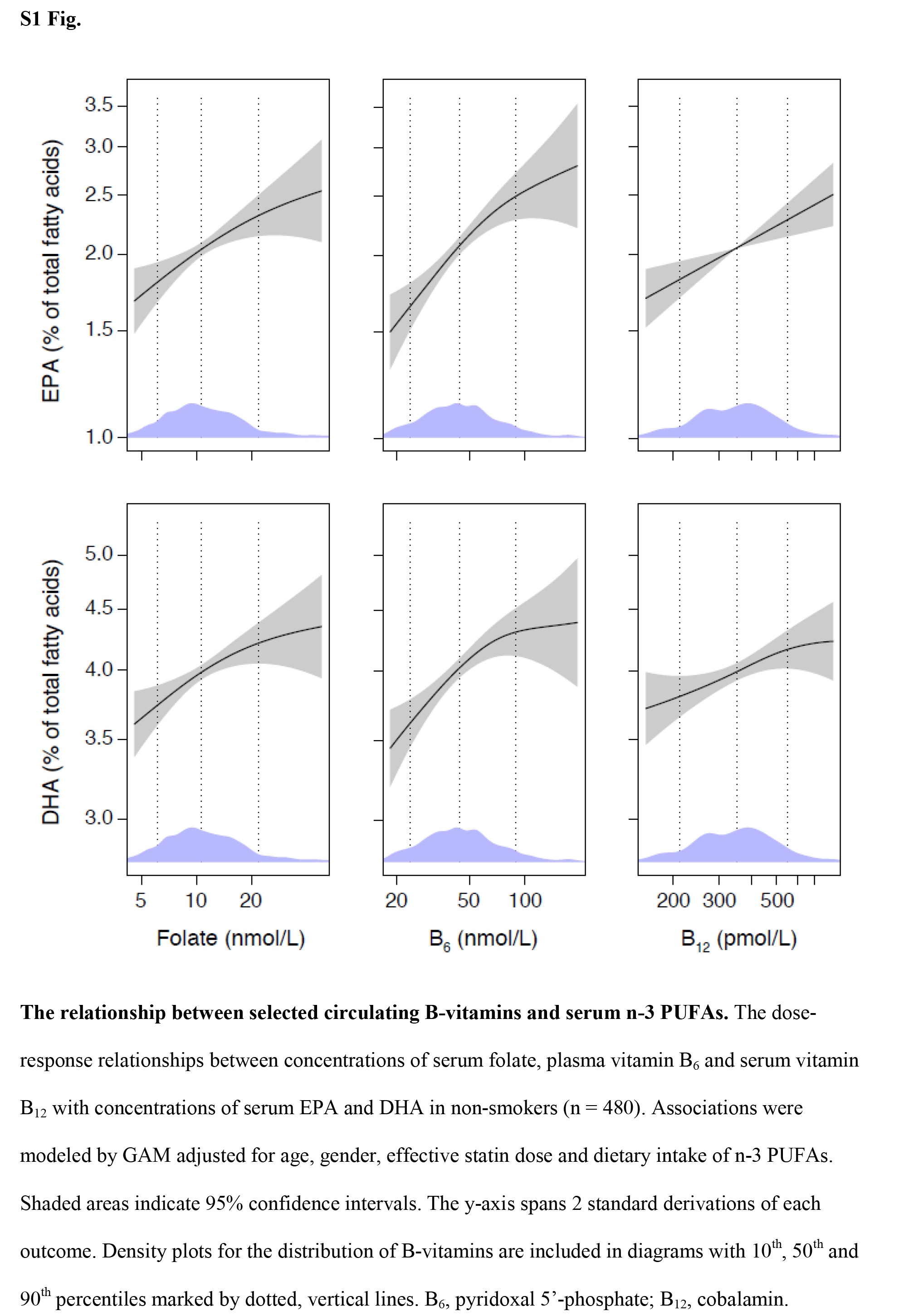

Supplement: S1 Fig — The dose-response relationships between concentrations of serum folate, plasma vitamin B6 and serum vitamin B12 with concentrations of serum EPA and DHA in non-smokers (n = 480). Associations were modeled by GAM adjusted for age, gender, effective statin dose and dietary intake of n-3 PUFAs. Shaded areas indicate 95% confidence intervals. The y-axis spans 2 standard derivations of each outcome. Density plots for the distribution of B-vitamins are included in diagrams with 10th, 50th and 90th percentiles marked by dotted, vertical lines. B6, pyridoxal 5’-phosphate; B12, cobalamin. (TIF) [file pone.0129049.s001.tif]

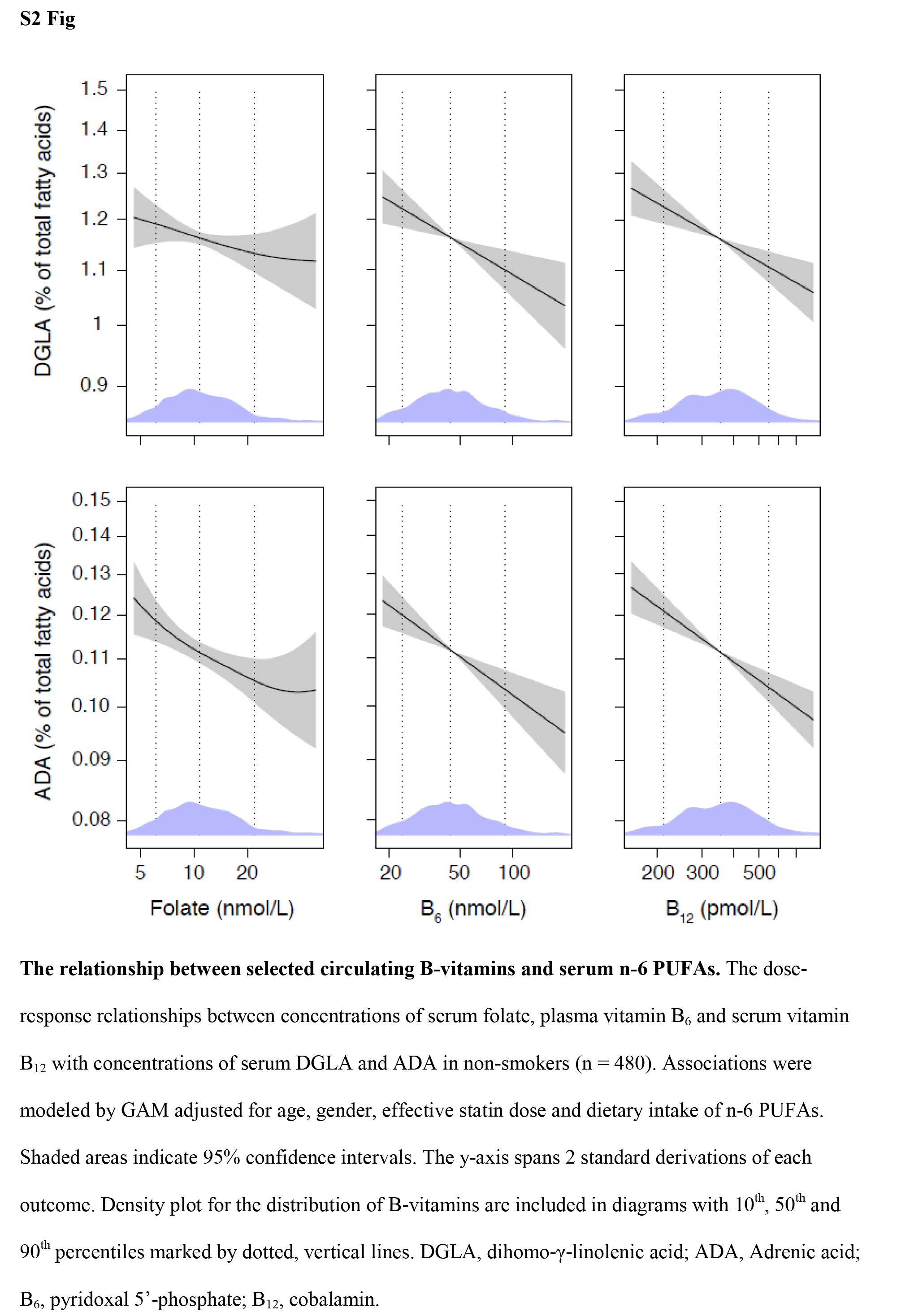

Supplement: S2 Fig — The dose-response relationships between concentrations of serum folate, plasma vitamin B6 and serum vitamin B12 with concentrations of serum DGLA and ADA in non-smokers (n = 480). Associations were modeled by GAM adjusted for age, gender, effective statin dose and dietary intake of n-6 PUFAs. Shaded areas indicate 95% confidence intervals. The y-axis spans 2 standard derivations of each outcome. Density plot for the distribution of B-vitamins are included in diagrams with 10th, 50th and 90th percentiles marked by dotted, vertical lines. DGLA, dihomo-γ-linolenic acid; ADA, Adrenic acid; B6, pyridoxal 5’-phosphate; B12, cobalamin. (TIF) [file pone.0129049.s002.tif]

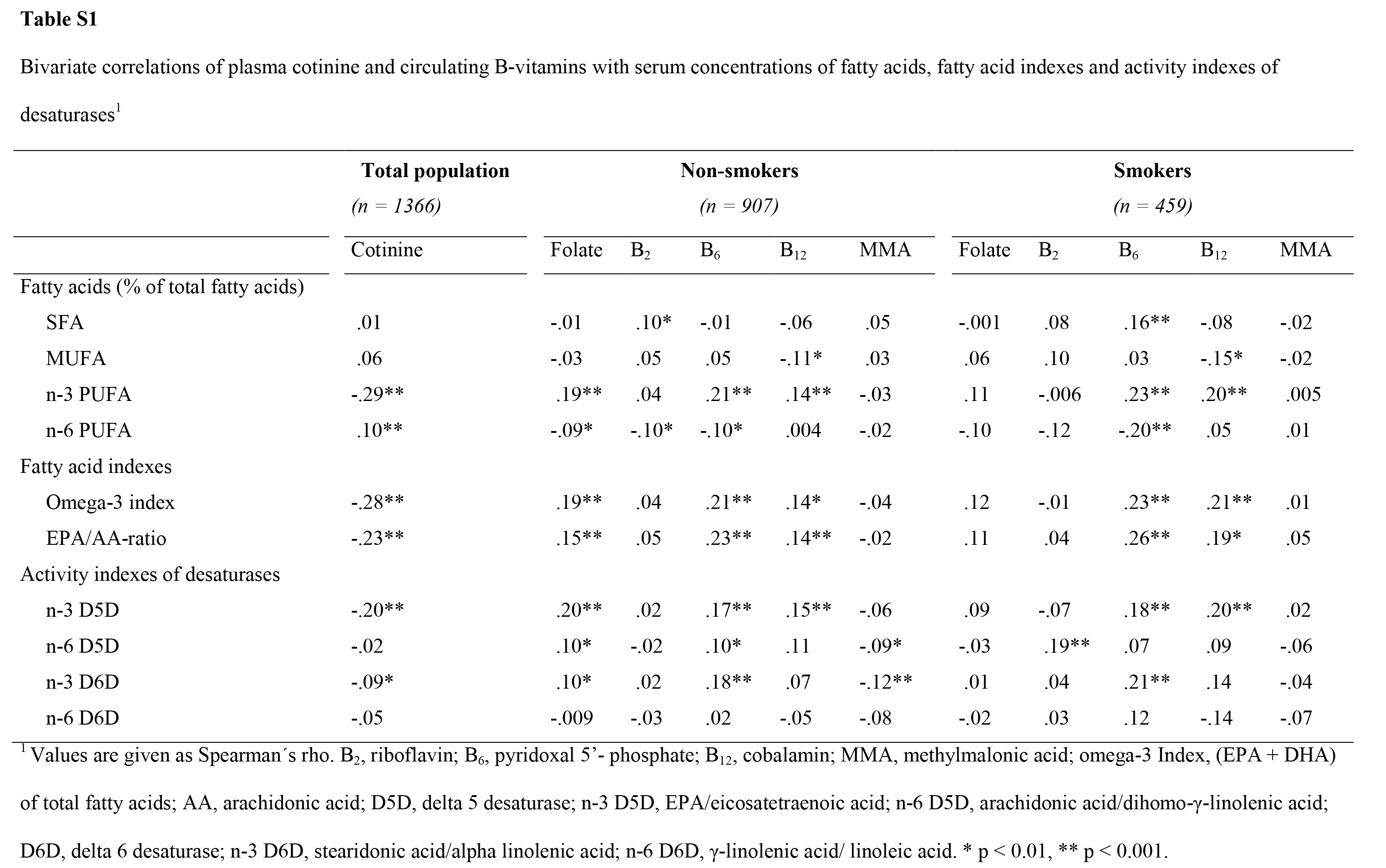

Supplement: S1 Table — 1 Values are given as Spearman’s rho. B2, riboflavin; B6, pyridoxal 5’- phosphate; B12, cobalamin; MMA, methylmalonic acid; omega-3 Index, (EPA + DHA) of total fatty acids; AA, arachidonic acid; D5D, delta 5 desaturase; n-3 D5D, EPA/eicosatetraenoic acid; n-6 D5D, arachidonic acid/dihomo-γ-linolenic acid; D6D, delta 6 desaturase; n-3 D6D, stearidonic acid/alpha linolenic acid; n-6 D6D, γ-linolenic acid/ linoleic acid. * p < 0.01, ** p < 0.001. (TIF) [file pone.0129049.s003.tif]
